# Liraglutide improves depressive and cognitive deficits in a high-fat diet rat model of obesity: the role of hippocampal autophagy and the PI3K/Akt/mTOR pathway

**DOI:** 10.1007/s00213-025-06834-7

**Published:** 2025-06-17

**Authors:** Yosra M. Magdy, Sherif A. Kamar, Mohamed Z. Habib, Hagar Yousry Rady, Mohammed R. Rabei, Sara Khedr

**Affiliations:** 1 Department of Clinical Pharmacology, Faculty of Medicine, Ain Shams University, Abbassia, Cairo, 11566 Egypt; 2 Department of basic medical sciences, Faculty of Dentistry, Al-Ahliyya Amman University (AAU), Amman, Jordan; 3 Department of Anatomy and Embryology, Faculty of Medicine, Ain Shams University, Cairo, Egypt; 4 Department of Basic Medical Sciences, Faculty of Medicine, King Salman International University, El Tor, South Sinai Egypt; 5 Department of Anatomy and Embryology, Armed Forces College of Medicine (AFCM), Cairo, Egypt; 6 Department of Medical Physiology, Faculty of Medicine, Mansoura University, Mansoura, Egypt

**Keywords:** Depression, High-fat diet, Liraglutide, Cognitive dysfunction, Autophagy, Hippocampus

## Abstract

**Rationale:**

Psychiatric disorders are a largely elusive aspect of obesity, representing a growing public health concern. In this regard, a large body of evidence indicates a pivotal role of disturbed autophagic flux in the pathogenesis of obesity-associated neuropsychiatric deficits.

**Objectives:**

This work was designed to evaluate the effects of the glucagon-like peptide-1 (GLP-1) receptor agonist liraglutide, which is increasingly utilized for the management of chronic obesity, on the depressive/cognitive deficits in the high-fat diet (HFD) rat model of obesity with an emphasis on its hippocampal mechanistic backgrounds.

**Methods:**

The effects of chronic liraglutide administration (subcutaneous; 300 µg/kg/day for 28 days) were investigated on depressive-like phenotypes, cognitive deficits, and hippocampal phosphatidylinositol 3-kinase (PI3K)/protein kinase B (Akt)/mechanistic target of rapamycin (mTOR)-regulated autophagy.

**Results:**

Chronic liraglutide treatment amended the HFD-induced depressive-like phenotype (in the sucrose preference and the forced swimming tests) and cognitive deficits (in the Morris water maze test). Moreover, liraglutide enhanced the hippocampal expression of brain-derived neurotrophic factor (BDNF), PI3K, Akt, p-Akt, and p-mTOR and downregulated the expression of the autophagic markers (Beclin-1, LC3) and the inflammatory markers (TNF-α, IL-6) with amelioration of HFD-induced hippocampal neurodegeneration.

**Conclusions:**

This work highlights the antidepressant and pro-cognitive properties of liraglutide in HFD-exposed rats, which could be mediated through amelioration of the disrupted PI3K/Akt/mTOR signaling activity with a possible impedance of the exaggerated autophagy-mediated neurodegenerative cascades. Indeed, this study highlights that liraglutide is not only effective in weight control, but its effects also extend to managing obesity-related psychiatric disorders.

## Introduction

Obesity and depression are very interconnected; each disease represents a major risk factor for the other. It is no wonder that the incidence of both conditions has increased steadily over the past few years (Fulton et al. 2022). Indeed, a wide array of mechanisms could underlie the complex relationship between obesity and depression, including poor dietary habits and physical inactivity, visceral fat accumulation with subsequent metabolic alterations, vascular dysfunction, insulin resistance, and inflammation, which in turn could disrupt neuroimmune status and neuronal connectivity, ultimately leading to mood disorders including depression (Fulton et al. 2022; Fu et al. 2023).

Macro-autophagy (referred to as autophagy) is a well-conserved cell survival mechanism involved in the removal of damaged cytosolic organelles and misfolded proteins to maintain cellular homeostasis. During autophagy, target cellular substrates are packaged in a double-membrane vesicle (autophagosome) that fuses with lysosomes for degradation of its contents (King 2012; Tang et al. 2021).

The phosphatidylinositol 3-kinase (PI3K)/protein kinase B (Akt) pathway represents an important upstream activator of the mechanistic target of rapamycin (mTOR) (Wu et al. 2009; Zhang et al. 2020). In turn, and through the formation of the mTOR complex 1 (mTORC1), the active phosphorylated mTOR acts as a major inhibitory regulator of autophagy (Deleyto-Seldas and Efeyan 2021). Interestingly, impaired autophagy is considered an important pathologic consequence associated with obesity (Behrooz et al. 2024). Moreover, decreased PI3K/Akt/mTOR signaling with dysfunctional autophagy has been intensely linked to hippocampal neuronal damage and subsequent depressive-like behaviors and cognitive dysfunction (Shih et al. 2019; Zhang et al. 2021; Xu et al. 2023).

Liraglutide is a glucagon-like peptide-1 (GLP-1) agonist used for the treatment of diabetes mellitus. In addition to its favorable effects on glucose homeostasis, liraglutide is thought to improve hypertension, lipid profiles, and obesity (Nikolic et al. 2022). Subcutaneous liraglutide is indicated as an adjunct to diet regulation and physical exercise for the management of chronic obesity in adults with a BMI ≥30 kg/m^2^ (Cena et al. 2020). In addition to its metabolic effects, liraglutide was shown to exhibit neuroprotective, antidepressant, and pro-cognitive effects in human subjects and in rodents as well (Weina et al. 2018; Kahal et al. 2019; Guan et al. 2023; Seo et al. 2023). Indeed, activation of the PI3K/Akt/mTOR signaling pathway and subsequent autophagic modulation could underlie the favorable antidepressant and pro-cognitive actions of liraglutide (Wang et al. 2018; Yang et al. 2018; Yao et al. 2021; Guan et al. 2023).

This study was designed to evaluate the effects of liraglutide on hippocampal PI3K/Akt/mTOR signaling and autophagy and its role in the alleviation of high-fat diet (HFD)-induced depressive-like behaviors and cognitive deficits. To the best of the authors’ knowledge, this study could be the first to investigate the role of liraglutide in autophagic modulation in the brains’ of HFD-exposed rats.

## Materials and methods

### Animals

Ninety-six adolescent male Wistar rats weighing 150–180 g (6 weeks of age) were purchased from the Holding Company for Biological Products and Vaccines (VACSERA, Helwan, Egypt) and were given a one-week acclimatization period before undergoing any experiments. Rats were housed in groups in plexiglass cages with mesh wire covers (4 rats per cage). Animals were maintained at a constant temperature of 24 ± 1 °C and a 12-hour light/dark cycle from 5:00 a.m. to 5:00 p.m. with good ventilation. Food and water were provided without restriction, unless otherwise recommended by the study protocol. The cages were cleaned daily throughout the course of the experiment. All efforts were made to minimize animal suffering as well as reduce the number of animals used. The study was conducted in accordance with EU Directive 2010/63/EU for animal experiments and was approved by the Research Ethics Committee of the Faculty of Medicine, Ain Shams University (FMASU-REC). FMASU-REC operates under Federal Wide Assurance. No. FWA 000017585 (approval number: FMASU R231/2022).

###  High-fat diet obesity model

**Fig. 1:**
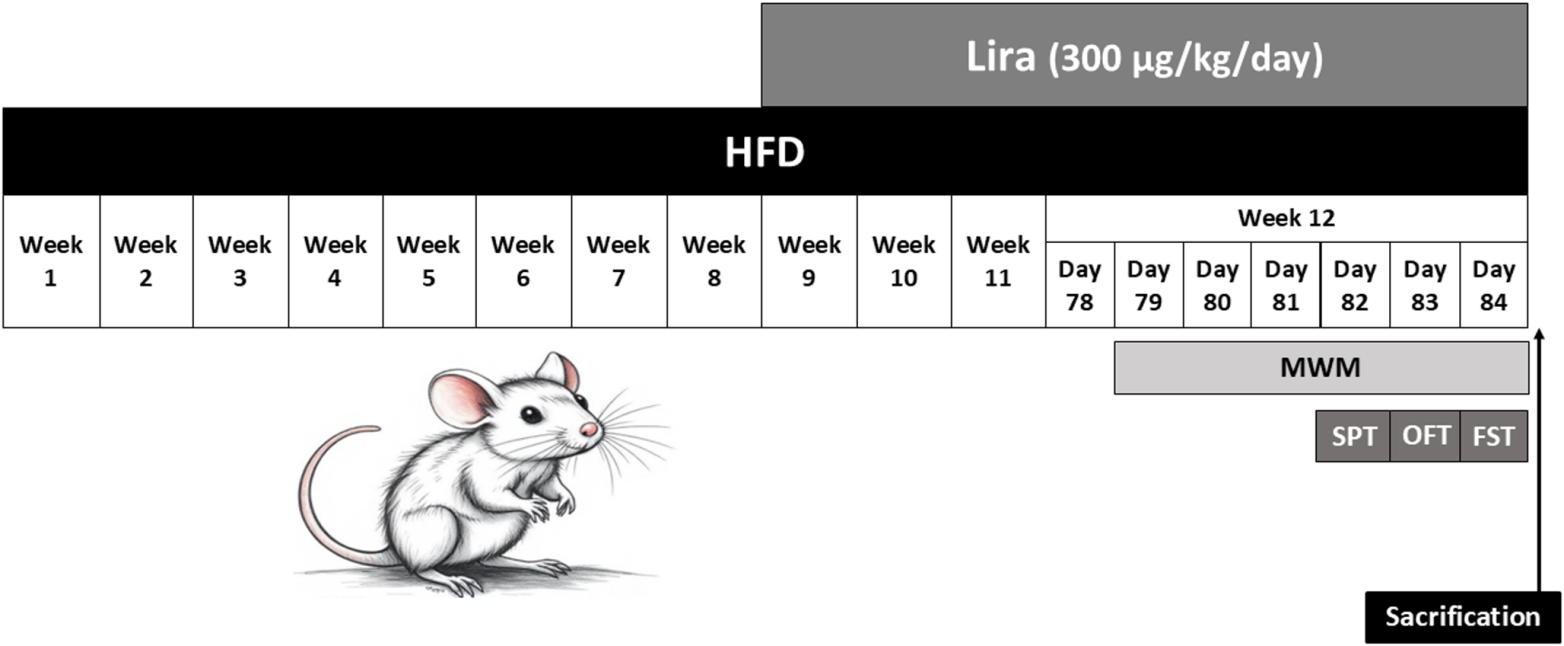
A diagram showing the experimental exposures of the study. Abbreviations: Lira, Liraglutide; HFD, High-fat diet; MWM, Morris water maze test; SPT, Sucrose preference test; OFT, Open field test; FST, Forced swimming test

Rats were fed a high-fat diet (HFD) for 12 weeks. The HFD consisted of 72.8% regular chow (containing approximately 20% proteins, 10% fat, and 70% carbohydrates), 2% cholesterol, 0.2% bile acids, and 25% lard (Magdy et al. 2017). The regular chow used in this study was sourced from Meladco for animal food (El-Obour, Al-Qalyubia, Egypt). The experimental exposures of the study are shown in Fig. 1.

### Experimental groups

The rats were randomly divided into four equal groups (24 rats each) using GraphPad StatMate, Software Inc., Version 1.01i (1998), CA, USA:

- *Control group*: rats were fed a regular chow diet and received daily subcutaneous (s.c.) injections of saline over the last 4 weeks of the experiment.
- *Lira group*: rats were fed a regular chow diet and received daily s.c. injections of liraglutide (Victoza, Novo Nordisk, Bagsvaerd, Denmark) at a dose of 300 µg/kg/day (Palleria et al. 2017) over the last 4 weeks of the experiment.
- *HFD group*: rats were fed a HFD and received daily s.c. injections of saline over the last 4 weeks of the experiment.
- *HFD/Lira group*: rats were fed a HFD and received daily s.c. injections of liraglutide at a dose of 300 µg/kg/day over the last 4 weeks of the experiment.

### Body weight and behavioral tests

Animal body weights were assessed at the beginning of the study and weekly thereafter. Behavioral testing was conducted during the last week of the study. Twelve rats per group were tested for the Morris water maze test; these rats were not exposed to other behavioral tests. The remaining rats were exposed to the sucrose preference test, the open field test, and then the forced swimming test. During behavioral testing and histological and biochemical measurements, trained observers were blinded to treatments. Behavioral tests were conducted on separate days to reduce the impact of sequential testing. Treatments were received after the completion of the behavioral testing on the corresponding day. Animals were sacrificed the next day in the morning.

#### Sucrose preference test (SPT)

Rats were trained to distinguish between tap water and a 1% sucrose solution in two distinct bottles for a week before the test. Rats were allowed to freely choose between two 200-ml bottles during the test: one contained tap water, and the other contained a 1% sucrose solution. By calculating the weight difference between the bottles before and after the 24-hour period, the amounts of water and sucrose solution consumed were calculated. The proportion of sucrose solution consumed out of the total amount of fluids was then used to calculate the sucrose preference using the following formula:

$$Sucrose\;preference\;\left(\%\right)=\left(sucrose\;intake/total\;fluid\;intake\right)\;\times\;100\%.$$

To prevent getting accustomed to the sucrose side during training and testing, the bottles’ positions were swapped every time (Mohamed et al. 2020).

#### Open field test (OFT)

Each rat was placed individually in the center of a square, quadrangular arena measuring 60 × 60 × 45 cm. Black lines divided the arena’s floor into 16 identical squares. The rats were given five minutes to explore the arena, during which their behavior was videotaped for later manual analysis. The number of crossed squares (squares crossed with all four paws), frequency of rearing, time spent in the central zone (central four squares), frequency of entering the central zone, and latency to leave the central zone were measured. To ensure that the rats were accustomed to the testing environment, they were allowed to acclimatize to the test room for 1 h before the start of the experiment. After each test session, the arena was cleaned using a 10% alcohol solution to eliminate any potential olfactory cues that could influence the behavior of the subsequent rats. The test was conducted in a well-ventilated, darkened, and sound/light-attenuated testing room (Habib et al. 2023).

#### Forced swimming test (FST)

During the FST, rats were allowed to swim in a vertical Plexiglass cylinder (diameter 22.5 cm, height 50 cm) filled with water (maintained at 25 ˚C) to the level of 35 cm. On the 1 st day (training), rats were trained to swim for 15 min, then removed from the cylinder and allowed to dry before they were returned to their home cages. On the 2nd day (test), rats were allowed to swim for 5 min. Rat’s behavior was videotaped, and the immobility time (slight activity where the animals just make movements necessary to keep their heads above the water surface) was calculated. The test was performed on each rat only once. Water was changed after testing each animal (Mohamed et al. 2020).

#### Morris water maze (MWM)

The Morris water maze was used to test spatial learning and memory in rodents, particularly in relation to hippocampal functioning. A hidden platform with a diameter of 10 cm was buried 1 cm under the water’s surface in a circular pool with a diameter of 1.8 m filled with water (25 °C) to a depth of 40 cm. During the spatial training, rats were placed in the water at various starting positions, and they had to navigate to find the hidden platform. In order to provide the rats with spatial references, visual cues like posters or objects were put in strategic locations throughout the testing space. The rats’ movements were recorded using a video camera mounted on the ceiling above the pool. Over the course of five days, the rats underwent four trials per day. In each trial, a single rat was gently placed into the water from alternating starting positions, facing the edge of the pool, and the time spent to reach the platform was recorded. If a rat failed to reach the platform within 90 s, it was gently guided to the platform and allowed to remain on it for 15 s. The daily average time spent to reach the platform was calculated. Twenty-four hours after the last training session, a thirty-second probe trial was conducted, during which the submerged platform was removed and the trial was started from a new starting position. The time spent in the target quadrant (which formerly contained the platform) was presented as a percentage of the total trial length (Zohny et al. 2023).

### Sacrification and sample collection

Twenty-four hours after the last liraglutide injection, rats were anaesthetized using an intraperitoneal injection of urethane at a dose of 1.2 g/kg (Raafat et al. 2023). Retro-orbital collection of blood was done, and serum was separated and stored for biochemical measurements. For the biochemical assays (6 rats per group), trans-cardiac perfusion through the left ventricle was performed with 40 ml of phosphate-buffered saline (PBS) (pH 7.4), and then decapitation was done. Rats’ brains were dissected on ice-cold plates, and the hippocampus was rapidly collected and immediately frozen (−80 ℃). For the histological study (6 rats per group), trans-cardiac perfusion was performed with 40 ml of 10% neutral buffered formalin, then the head was decapitated, and the brain was dissected and fixed in 10% neutral buffered formalin (pH 7.4). Parasagittal cuts were made to all fixed specimens, and then the samples were processed and embedded into paraffin blocks. For the electron microscopy study (6 rats per group), transcardiac perfusion was done with 40 ml of 2.5% phosphate buffered glutaraldehyde (pH 7.4), then the head was decapitated, and the hippocampus was dissected out (Habib et al. 2022).

### Biochemical and molecular studies

#### Serum corticosterone

Serum corticosterone was measured using the corticosterone ELISA kit (cat# EIA-5186; DRG, Marburg, Germany) according to the manufacturer’s instructions. Absorbance was measured at 450 nm.

#### Gene expression of *PI3K*, *Akt*, *mTOR*,* Beclin 1*, and *LC3*

Gene expression of *PI3K*, *Akt*, *mTOR*,* Beclin 1*, and *LC3* was estimated in hippocampal tissue homogenates using real-time quantitative polymerase chain reaction (RT-qPCR) analysis. Direct-zol RNA Miniprep Plus (cat# R2072; Zymo Research, Irvine, CA, USA) was used for the extraction of total RNA from homogenized hippocampal samples according to the manufacturer’s instructions. Reverse transcription of the extracted RNA was done using the SuperScript™ IV One-Step RT-PCR kit (Cat# 12594100, Thermo Fisher Scientific, Waltham, MA, USA), followed by PCR. StepOne instrument (Applied Biosystem, Waltham, MA, USA) was utilized in a thermal profile as follows: 45 ºC for reverse transcription (10 minutes), 98 ºC for RT inactivation (2 minutes), and initial denaturation by 40 cycles of 10 seconds at 98 ºC, 10 seconds at 55 ºC, and 30 seconds at 72 ºC for the amplification step. The data was expressed as a cycle threshold (Ct) for the target genes and housekeeping gene. For *Beclin 1*, the forward primer sequence was 5′-CTCTCGTCAAGGCGTCACTTC-3′ and the reverse primer sequence was 5‵-CCTTAGACCCCTCCATTCCTCA-3′. For *LC3*, the forward primer sequence was 5′-CCTGCTGCTGGCCGTAGT-3′ and the reverse primer sequence was 5‵-TGATGAAGTCTTCCTGCCAAAA-3′. For *PI3K*, the forward primer sequence was 5′-CTCTCCTGTGCTGGCTACTGT-3′ and the reverse primer sequence was 5‵-GCTCTCGGTTGATTCCAAACT-3′. For *Akt*, the forward primer sequence was 5′-ATCCCCTCAACAACTTCTCAGT-3′ and the reverse primer sequence was 5‵-CTTCCGTCCACTCTTCTCTTTC-3′. For *mTOR*, the forward primer sequence was 5′-GGTGGACGAGCTCTTTGTCA-3′ and the reverse primer sequence was 5‵-AGGAGCCCTAACACTCGGAT-3′. *β-actin* was used as the reference gene; the forward primer sequence was 5’-TCTTCCAGCCTTCCTTCCTG-3’ and the reverse primer sequence was 5’-GATCACGAGGTCAGGAGATG-3’. The 2^− ΔΔCt^ method was conducted for the analysis of gene expression levels (Livak and Schmittgen 2001).

#### Protein levels of PI3K, akt, p-Akt, BDNF, mTOR, p-mTOR, Beclin 1, LC3, TNF-α, and IL-6

Hippocampal PI3K, Akt, p-Akt, BDNF, mTOR, p-mTOR, Beclin 1, LC3, TNF-α, and IL-6 were measured using the rat PI3K ELISA kit (cat# MBS260381; MyBioSource, San Diego, CA, USA), the total Akt + Akt (pS473) ELISA kit (cat# ab126433; Abcam, Cambridge, UK), the rat BDNF ELISA kit (cat# KT-8575; Kamiya Biomedical Co., Tukwila, WA, USA), the rat mTOR ELISA kit (cat# MBS744326; MyBioSource, San Diego, CA, USA), the phospho-mTOR (Ser2448) ELISA kit (cat#7976; Cell Signaling Technology, Danvers, MA, USA), the rat Beclin 1 ELISA kit (cat# NBP2-69960; Novus Biologicals, Centennial, CO, USA), the rat LC3B ELISA kit (cat# MBS1600540; MyBioSource, San Diego, CA, USA), the rat TNF-α ELISA kit (cat# SRTA00; Quantikine^®^, R&D Systems, Minneapolis, MN, USA), and the rat IL-6 ELISA kit (cat# MBS2021530; MyBioSource, San Diego, CA, USA) according to the manufacturer’s instructions. Absorbance was measured at 450 nm. The Bradford technique was utilized for measuring each sample’s protein concentration (Bradford 1976).

### Histological studies

#### Light microscopic study

The blocks were cut into 4 μm paraffin sections by a rotator microtome. The sections were deparaffinized and stained with Hematoxylin and Eosin (H&E) (Hasan et al. 2022; Abdullah et al. 2024). All sections were examined and photographed using a light microscope (Olympus 268 M; Olympus Optical Co., Shinjuku, Tokyo, Japan) and photographed using a digital camera (Nikon, Tokyo, Japan).

#### Electron microscopic study

For three hours at 4 °C, 1 mm^3^ thick brain sections were fixed in 3% phosphate buffered glutaraldehyde with a pH of 7.3. After two rinses in phosphate buffer for four hours, the specimens were post-fixed in 1% buffered osmium tetroxide (1–2 h) at a temperature of 4 °C. After that, the tissue fragments were rinsed twice in phosphate buffer for 30 min each. Following that, the specimens were dehydrated in ascending grades of ethyl alcohol (50%, 70%, 80%, 90%, and absolute alcohol) for 15 min twice in each grade but 30 min in absolute alcohol. Propylene oxide was used for clearing for 20 min at room temperature. Infiltration was then performed overnight using equal parts of propylene oxide and Epon-812. Finally, the specimens were placed into gelatin capsules containing fresh Epon. To allow polymerization, the capsules were heated in a 60 °C oven for 48 h (Bancroft and Gamble 2008). Ultrathin sections, 50–80 nm, were chosen from the blocks on the ultramicrotome, placed on copper grids, and counterstained with uranyl acetate and lead citrate (Deng et al. 2019). These sections were investigated using the transmission electron microscope (TEM) (JEM-1010; JEOL Ltd., Tokyo, Japan) at the Electron Microscope Unit, Faculty of Science, Ain Shams University.

### Statistical analysis

All values were expressed as the mean ± S.E.M. Statistical analysis was carried out using the GraphPad Prism software program, version 8.0.1 (2018), Inc., CA, USA. The data was tested for normality using the D’Agostino and Pearson omnibus normality test. The statistical difference among groups was determined using a two-way ANOVA followed by Tukey’s *post-hoc* test for comparisons between more than two groups. A repeated-measures ANOVA was utilized for the analysis of body weight and time to reach the platform in MWM. *P* values < 0.05 were considered statistically significant.

## Results

### Body weight

**Fig. 2:**
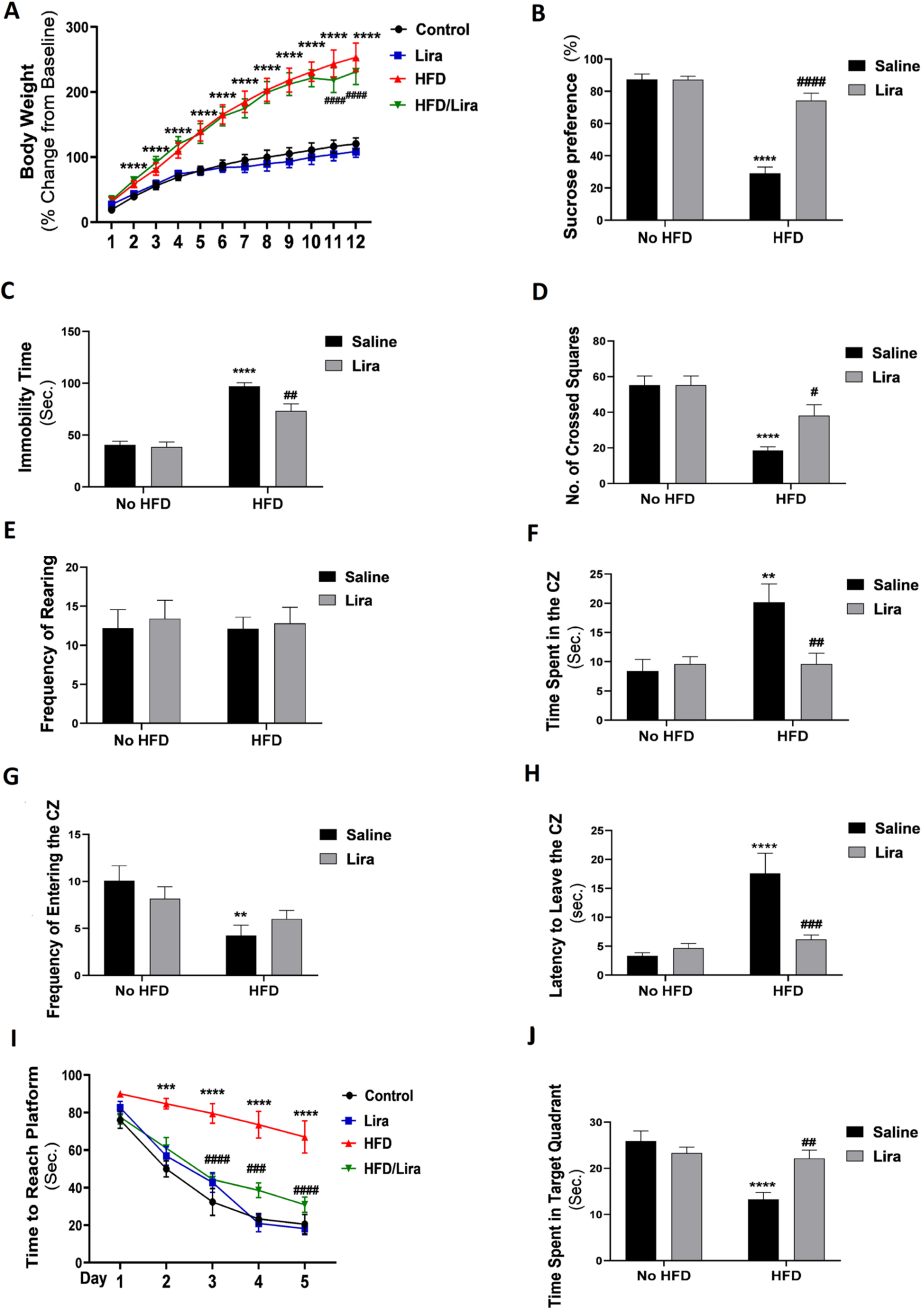
Effects of liraglutide on body weight change, sucrose preference, forced swimming test immobility time, open field test, and the Morris water maze (MWM) behavioral changes in HFD-exposed Wistar rats. Data are presented as mean ± S.E.M. (*n* = 12 except for the body weight, *n* = 24). ^**^P < 0.01, ^***^P < 0.001, ^****^P < 0.0001 vs. Control group; ^#^*P* < 0.05, ^##^*P* < 0.001, ^###^*P* < 0.001, ^####^*P* < 0.0001 vs. HFD group. Treatments were compared by a two-way ANOVA followed by Tukey’s post-hoc test. For the body weight change and the latency to reach the MWM hidden platform, treatments were compared by a repeated-measures ANOVA followed by Tukey’s post-hoc test

As shown in Fig. 2A, repeated-measures ANOVA revealed a significant interaction between HFD, Lira, and time (F _(11, 1104)_ = 1.807, *P* < 0.05). HFD induced a significant increase in body weight starting from the end of the 2nd week to the end of the study (*P* < 0.0001) compared to the control group. Lira treatment induced a significant decline in body weight at the end of the 11th and 12th weeks (*P* < 0.0001) compared to the HFD group.

### Behavioral tests

#### Sucrose preference test

Two-way ANOVA revealed a significant interaction between HFD and Lira (F _(1, 44)_ = 40.08, *P* < 0.0001). The HFD group exhibited a significant decrease in sucrose preference (*P* < 0.0001) compared to the control group. Lira treatment induced a significant increase in sucrose preference (*P* < 0.0001) compared to the HFD group. (Fig. 2B).

#### Forced swimming test

As depicted in Fig. 2C, two-way ANOVA revealed a significant interaction between HFD and Lira (F _(1, 44)_ = 5.125, *P* < 0.05). The HFD group showed a significant increase in the immobility time (*P* < 0.0001) compared to the control group. The HFD/Lira group exhibited a significant decline in the immobility time (*P* < 0.01) compared to the HFD group.

#### Open field test

As shown in Fig. 2D-G, two-way ANOVA revealed a significant interaction between HFD and Lira (F _(1, 44)_ = 7.341, *P* < 0.01; F _(1, 44)_ = 7.423, *P* < 0.01 for the time spent in the central zone and the latency to leave the central zone respectively). The HFD group exhibited a significant decrease in the number of crossed squares (*P* < 0.0001) and the frequency of entry to the central zone (*P* < 0.01), with an increase in the central zone duration (*P* < 0.01) and the latency to leave the central zone (*P* < 0.0001) compared to the control group. The HFD/Lira group exhibited a significant increase in the number of crossed squares (*P* < 0.05) with a significant decrease in the central zone duration (*P* < 0.01) and the latency to leave the central zone (*P* < 0.001) compared to the HFD group. Notably, the study interventions did not induce significant effects on the frequency of rearing.

#### Morris water maze

As regards the time to reach the platform in the MWM test, repeated-measures ANOVA revealed a significant main effect of time (F _(4, 220)_ = 63.69, *P* < 0.0001), HFD (F _(1, 220)_ = 107.9, *P* < 0.0001), and Lira (F _(1, 220)_ = 32.76, *P* < 0.0001). The HFD group exhibited a significant increase in the time to reach the platform on the 2nd (*P* < 0.001), 3rd, 4th, and 5th (*P* < 0.0001) days compared to the control group. Lira treatment was able to significantly reverse HFD-induced effects on the 3rd (*P* < 0.0001), 4th (*P* < 0.001), and 5th (*P* < 0.0001) days compared to the HFD group (Fig. 2H).

As regards the probe trial, two-way ANOVA revealed a significant interaction between HFD and Lira (F _(1, 44)_ = 11.31, *P* < 0.01). The HFD group showed a significant decrease in the time spent in the target quadrant (*P* < 0.0001) compared to the control group. The HFD/Lira group exhibited a significant increase in the time spent in the target quadrant (*P* < 0.01) compared to the HFD group (Fig. 2I).

### Biochemical measurements

#### Gene expression of *PI3K*, *Akt*, *mTOR*,* Beclin 1*, and *LC3*

**Fig. 3:**
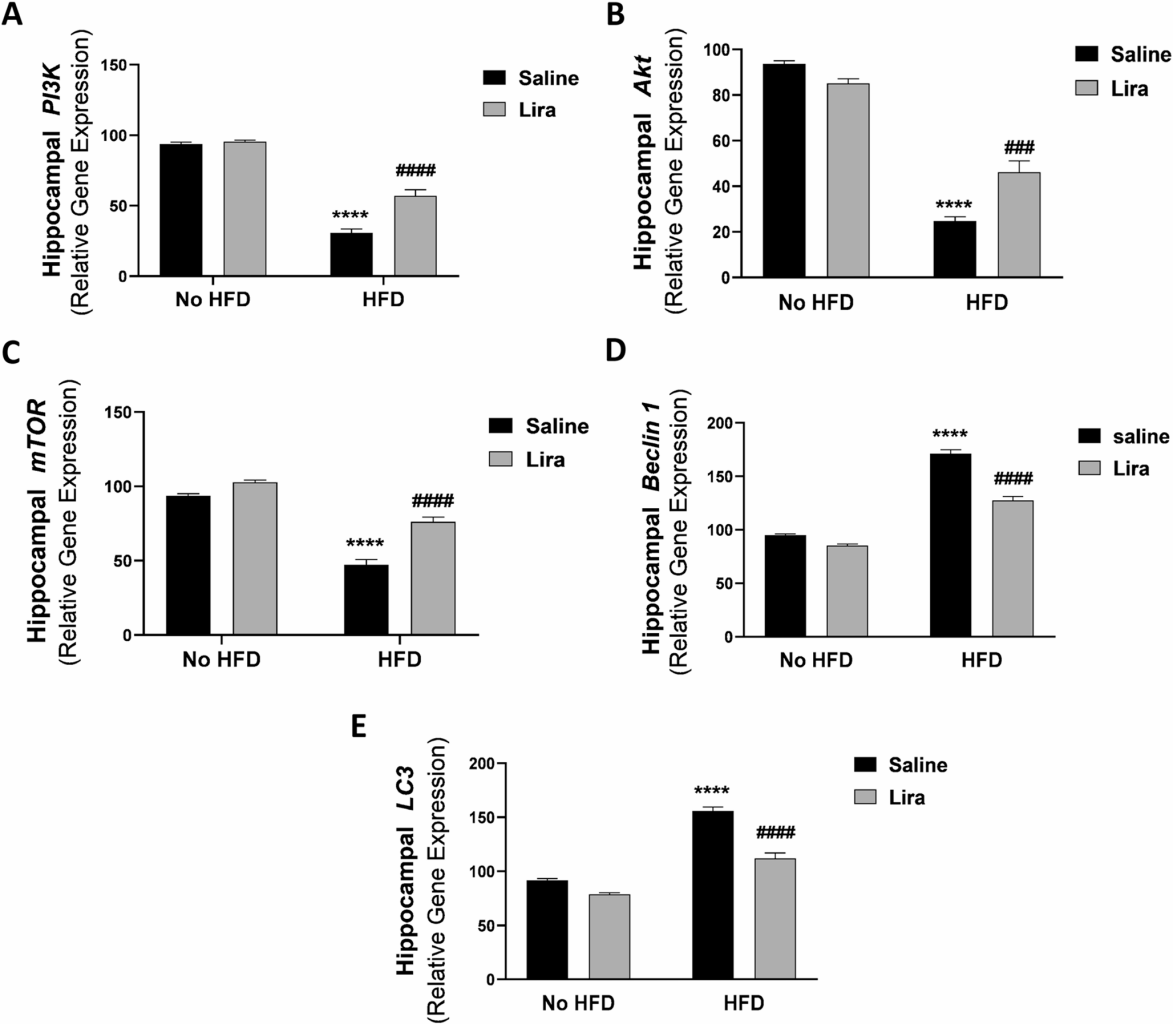
Effects of liraglutide on gene expression of *PI3K*, *Akt*, *mTOR*,* Beclin 1*, and *LC3* in hippocampal homogenates of HFD-exposed Wistar rats. Data are presented as mean ± S.E.M. (*n* = 6). ^****^P < 0.0001 vs. control group; ^###^*P* < 0.001, ^####^*P* < 0.0001 vs. HFD group. Treatments were compared by a two-way ANOVA followed by Tukey’s post-hoc test

As depicted in Fig. 3, two-way ANOVA revealed a significant interaction between HFD and Lira (F (1,20) = 20.10, *P* < 0.001; F (1,20) = 26.26, *P* < 0.0001; F (1,20) = 15.18, *P* < 0.001; F (1,20) = 37.78, *P* < 0.0001; F (1,20) = 23.54, *P* < 0.0001 for *PI3K*, *Akt*, *mTOR*,* Beclin 1*, and *LC3* respectively). The HFD group exhibited a significant decrease in hippocampal gene expression of *PI3K*, *Akt*, and *mTOR* (*P* < 0.0001) with an increase in hippocampal gene expression of *Beclin 1* and *LC3* (*P* < 0.0001) compared to the control group. The HFD/Lira group showed a significant increase in *PI3K* (*P* < 0.0001), *Akt* (*P* < 0.001), and *mTOR* (*P* < 0.0001) gene expression, alongside a decrease in the hippocampal gene expression of *Beclin 1* and *LC3* (*P* < 0.0001) compared to the HFD group.

#### PI3K, akt, p-Akt, BDNF, mTOR, p-mTOR, Beclin 1, and LC3 proteins

**Fig. 4:**
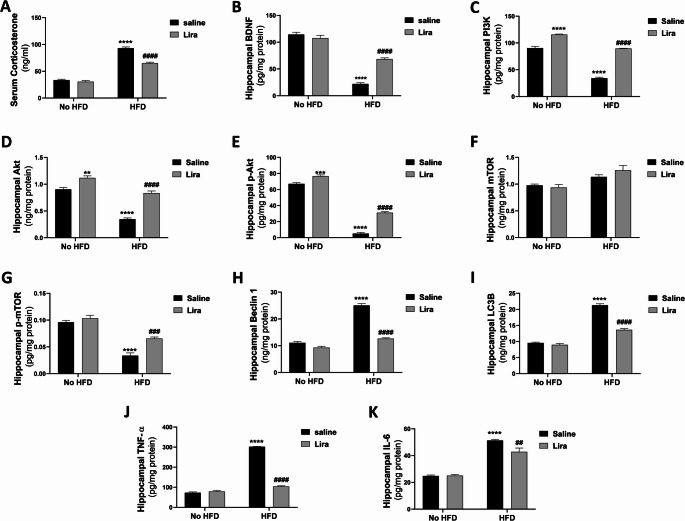
Effects of liraglutide on serum corticosterone and hippocampal BDNF, PI3K, Akt, p-Akt, mTOR, p-mTOR, Beclin 1, LC3B, TNF-α, and IL-6 in HFD-exposed Wistar rats. Data are presented as mean ± S.E.M. (*n* = 6). ^**^P < 0.01, ^***^P < 0.001, ^****^P < 0.0001 vs. control group; ^##^*P* < 0.01, ^###^*P* < 0.001, ^####^*P* < 0.0001 vs. HFD group. Treatments were compared by a two-way ANOVA followed by Tukey’s post-hoc test

As shown in Fig. 4, two-way ANOVA revealed a significant interaction between HFD and Lira (F (1,20) = 63.81, *P* < 0.0001; F (1,20) = 16.27, *P* < 0.001; F (1,20) = 32.28, *P* < 0.0001; F (1,20) = 52.31, *P* < 0.0001; F (1,20) = 7.754, *P* < 0.05; F (1,20) = 119.7, *P* < 0.0001; F (1,20) = 96.82, *P* < 0.0001 for PI3K, Akt, p-Akt, BDNF, p-mTOR, Beclin 1, and LC3B respectively). The HFD group exhibited a significant decrease in hippocampal proteins of PI3K, Akt, p-Akt, BDNF, and p-mTOR, with an increase in Beclin 1 and LC3B proteins (*P* < 0.0001) compared to the control group. The HFD/Lira group showed a significant increase in the hippocampal proteins of PI3K (*P* < 0.0001), Akt (*P* < 0.0001), p-Akt (*P* < 0.0001), BDNF (*P* < 0.0001), and p-mTOR (*P* < 0.001), alongside a decrease in Beclin 1 and LC3 proteins (*P* < 0.0001) compared to the HFD group.

#### Serum corticosterone and hippocampal TNF-α, and IL-6

As shown in Fig. 4, two-way ANOVA revealed a significant interaction between HFD and Lira (F _(1,20)_ = 42.11, *P* < 0.0001; F _(1,20)_ = 742.2, *P* < 0.0001; F _(1,20)_ = 7.871, *P* < 0.05 for serum corticosterone and hippocampal TNF-α, and IL-6 respectively). The HFD group exhibited a significant increase in serum corticosterone and hippocampal TNF-α and IL-6 (*P* < 0.0001) compared to the control group. In the HFD/Lira group, there was a significant decrease in serum corticosterone (*P* < 0.0001), hippocampal TNF-α (*P* < 0.0001), and IL-6 (*P* < 0.01) compared to the HFD group.

### Histological studies

#### Light microscopic study

**Fig. 5:**
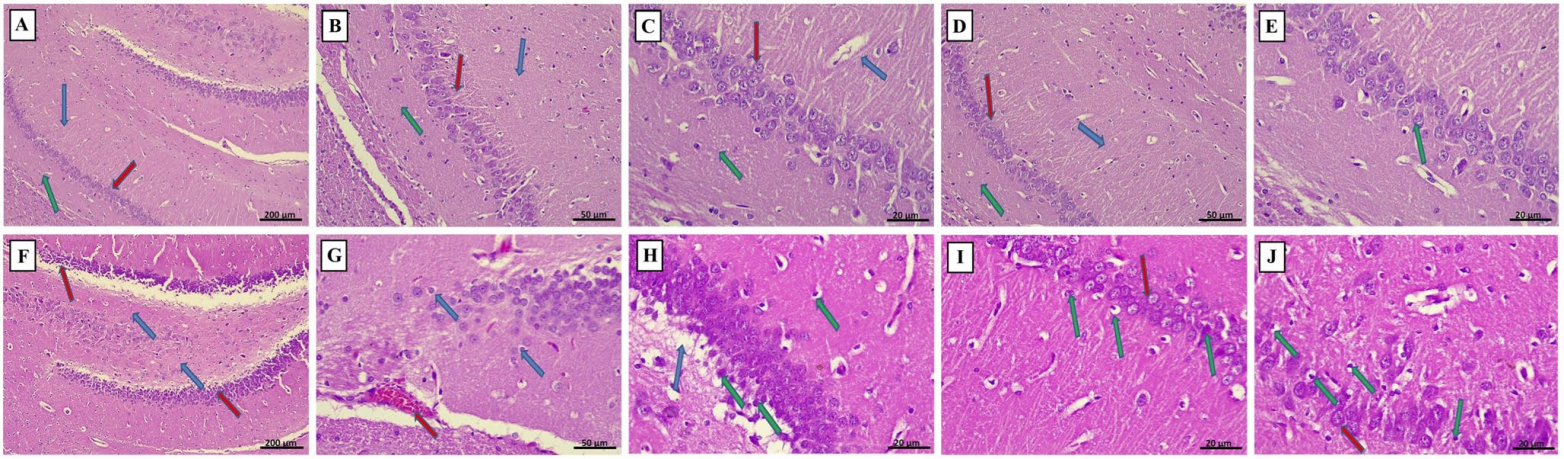
Photomicrographs of H&E-stained hippocampal sections of the control group (**A-C**), the Lira group (**D-E**), the HFD group (**F-H**), and the HFD/Lira group (**I-J**). The control group shows normal hippocampal structure, comprising the molecular layer (green ↑), the pyramidal cell layer (red ↑), and the polymorphic layer (blue ↑) ***(x100)*** (**A**). The molecular layer of the control group containing dendrites of the pyramidal cells (green ↑), the pyramidal cell layer containing the hippocampal pyramidal cells (red ↑), and the polymorphic layer containing many nerve fibers and small cell bodies of interneurons (blue ↑) ***(x200)*** (**B**). The molecular layer of the control group is formed of neurons characterized by a large cell body or perikaryon containing a large, pale active, euchromatic nucleus with a prominent nucleolus (green ↑), pyramidal cell layer (red ↑), and polymorphic layer (blue ↑) ***(x400)*** (**C**). Same findings are shown in the Lira group with normal hippocampal structure, comprising the molecular layer containing dendrites of the pyramidal cells (green ↑), the pyramidal cell layer containing hippocampal pyramidal cells (red ↑), and the polymorphic layer containing many nerve fibers and small cell bodies of interneurons (blue ↑) ***(x200)*** (**D**). The molecular layer of the Lira group is formed of neurons characterized by a large cell body or perikaryon containing a large, pale active, euchromatic nucleus with a prominent nucleolus (green ↑) ***(x400)*** (**E**). The HFD group shows pyramidal cell layer (red ↑) and polymorphic layer (blue ↑) degeneration with reduced thickness ***(x100)*** (**F**). Neuronal cell degeneration with irregular-shaped and pyknotic nuclei (blue ↑) and dilated congested blood vessels (red ↑) ***(x200)*** (**G**). Neuronal loss (blue ↑) and neuronal cell degeneration with irregular-shaped cell bodies, unclear cytoplasm, and pyknotic nucleus (green ↑) ***(x400)*** (**H**). The HFD/Lira group exhibits pyramidal cell layer exhibiting reduced thickness, yet some neurons appear to be normal (red ↑), others still degenerated (green ↑) ***(x400)*** (**I**,** J**)

H&E-stained sections of the control and Lira groups showed normal hippocampal structure in the form of the molecular layer containing dendrites of pyramidal cells, the pyramidal cell layer containing hippocampal pyramidal cells, and the polymorphic layer having many nerve fibers and small interneuron cell bodies. Neurons in the molecular layer possess large, pale, and active nuclei with prominent nucleoli. The HFD group exhibited degeneration and reduced thickness of both the pyramidal cell layer and the polymorphic layer. The molecular layer showed neuronal loss and further degeneration with irregular-shaped, pyknotic nuclei, irregular-shaped cell bodies, unclear cytoplasm, and dilated congested blood vessels. The HFD/Lira group showed reduced thickness of the pyramidal cell layer, with some neurons appearing normal while others remain degenerated (Fig. 5).

#### Transmission electron microscopy study

**Fig. 6:**
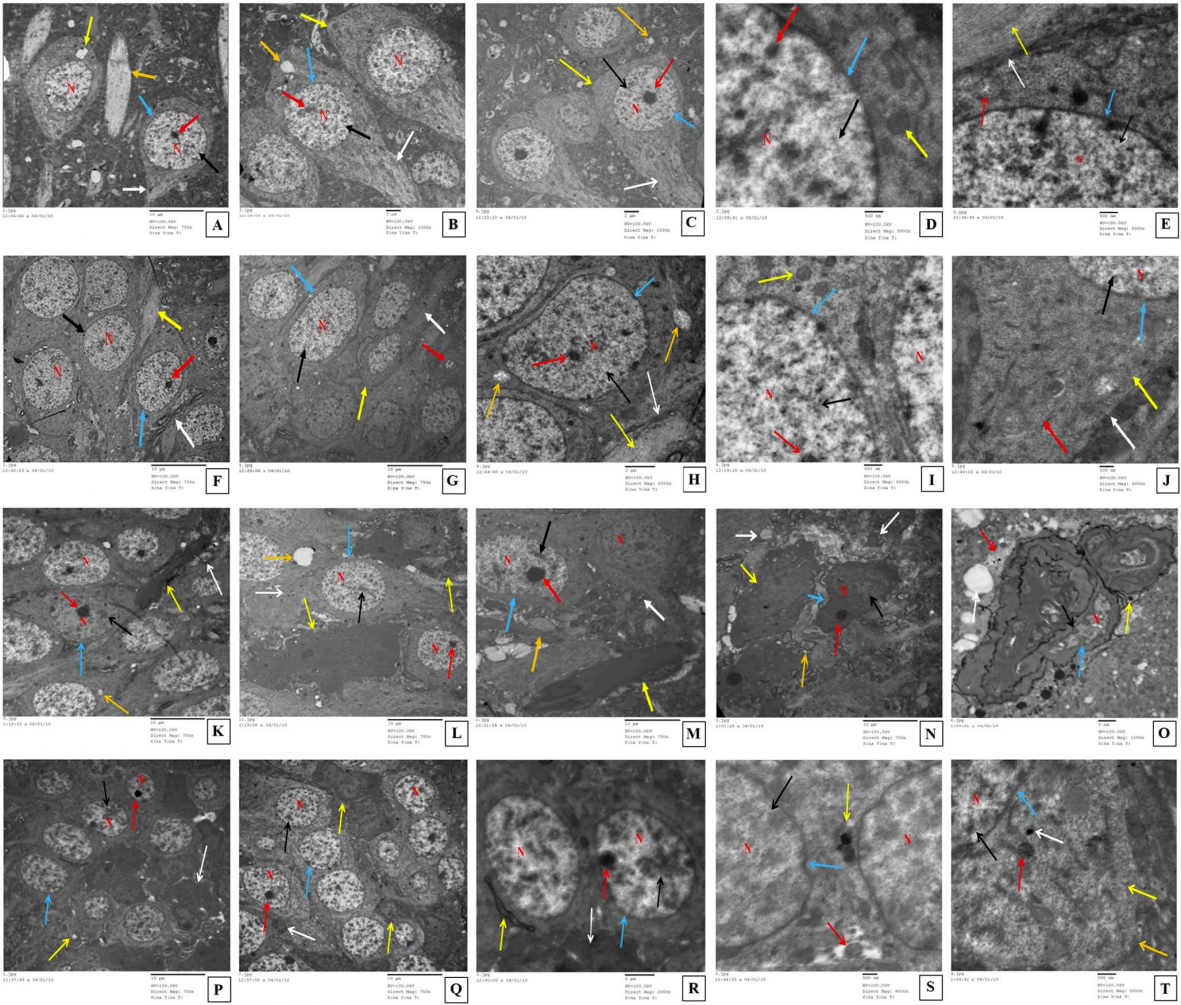
Photomicrographs of TEM-examined ultrathin hippocampal sections of the control group (**A-E**), the Lira group (**F-J**), the HFD group (**K-O**), and the HFD/Lira group (**P-T**). The control group shows neuronal cell bodies with nuclei (N) exhibiting dispersed chromatin (black ↑), prominent nucleoli (red ↑), regular nuclear membranes (blue ↑), regular basal dendrites (white ↑), regular apical dendrites (orange ↑), and small intra-cytoplasmic vacuoles (yellow ↑) (**A**,** B**,** C**). Higher magnifications showed neuronal cell bodies with nuclei (N) showing dispersed chromatin (black ↑), prominent nucleoli (red ↑), regular nuclear membranes (blue ↑), and average ovoid mitochondria (yellow ↑) (**D**), regular nuclear membranes (blue ↑), average axons (red ↑), portions of average regular dendrites (yellow ↑), and average junctions (white ↑) (**E**). The Lira group shows neuronal cell bodies with nuclei (N) exhibiting dispersed chromatin (black ↑), prominent nucleoli (red ↑), regular nuclear membranes (blue ↑), portions of average regular dendrites (yellow ↑), and average regular junctions (white ↑) (**F**). Basal (white ↑) and apical dendrites (yellow ↑) and average axons (red ↑) (**G**), average axons (orange ↑) (**H**). Higher magnifications showed neuronal cell bodies with nuclei (N) exhibiting dispersed chromatin (black ↑), prominent nucleoli (red ↑), regular nuclear membranes (blue ↑), and average ovoid mitochondria (yellow ↑) (**I**). Portions of average basal dendrites (yellow ↑), average axons (red ↑), and average regular junctions (white ↑) (**J**). The HFD group shows pyknotic nuclei (N) with clumped chromatin (black ↑), prominent nucleoli (red ↑), and regular nuclear membranes (blue ↑), electron-dense irregular dendrites (yellow ↑), autophagosomes with double layers (white ↑), and small intra-cytoplasmic vacuoles (orange ↑) (**K**,** L**), swollen mitochondria (orange ↑) (**M**), with higher magnification small condensed dendrites (orange ↑) (**N**), notched irregular nuclear membranes (blue ↑), and large vacuoles (white ↑) (**O**). The HFD/Lira group shows neuronal cell bodies with pyknotic nuclei (N) exhibiting clumped chromatin (black ↑), prominent nucleoli (red ↑), regular nuclear membranes (blue ↑), electron-dense irregular dendrites (white ↑), and small intra-cytoplasmic vacuoles (yellow ↑) (**P**), electron-dense regular dendrites (yellow ↑), average mitochondria (white ↑) (**Q**), and small, condensed dendrites (yellow ↑) (**R**). Higher magnification showed neuronal cell bodies with pyknotic nuclei (N) exhibiting dispersed chromatin (black ↑) and irregular nuclear membranes (blue ↑), average mitochondria (red ↑), and few autophagosomes with double layers (yellow ↑) (**S**) with scattered condensed mitochondria (red ↑), average dendrites (yellow ↑), few electron-dense bodies (white ↑), and average junctions (orange ↑) (**T**)

As shown in Fig. 6, the ultrathin sections of the control group and similarly the Lira group showed normal neuronal cell body structure with regular nuclear membrane, dispersed chromatin, prominent nucleoli, regular basal and apical dendrites and junctions, small intra-cytoplasmic vacuoles, average axons, and ovoid mitochondria with higher magnification. The HFD group exhibited signs of neuronal degeneration in the form of nuclear pyknosis and chromatin clumping, electron-dense irregular dendrites, small intra-cytoplasmic vacuoles, autophagosomes with double layers, and swollen mitochondria. Higher magnification revealed condensed dendrites and irregular nuclear membrane. Although the neuronal structure of the HFD/Lira group was apparently similar to the control and Lira groups, some neurons still showed signs of degeneration in the form of pyknotic nuclei and clumped chromatin, electron-dense irregular dendrites, and small intra-cytoplasmic vacuoles. Higher magnification showed autophagosomes, condensed mitochondria, and electron-dense bodies.

## Discussion

Obesity is often associated with various comorbidities, including mood disorders and cognitive impairments, which can significantly affect quality of life. This study highlights the potential of liraglutide, the GLP-1 receptor agonist, which is increasingly utilized for the management of chronic obesity, in addressing these neuropsychiatric challenges.

In the current work, HFD was associated with an increase in FST immobility time and a decrease in sucrose preference, which could infer behavioral despair and anhedonia, the core depressive symptoms (Cooper et al. 2018; Mohamed et al. 2020). The reduced frequency of entering the open field’s central zone infers thigmotaxis, an index of depression-associated anxiety (Gencturk and Unal 2024). The reduced number of crossed squares along with the increased latency to leave the open field’s central zone after placement in the center of the arena at the start of the test reflects the freezing behavior/hypoactivity, another index of depression-associated anxiety (Aboul-Fotouh 2013; Lezak et al. 2017). At first glance, it might be thought that the decreased number of crossed squares could represent a decrease in the general locomotor activity. However, the OFT square crossing not solely reflects motor capacity but also reflects the exploratory activity of rats, which could be impaired due to the reduced motivation to explore in the depressed rat. Notably, several studies reported HFD-induced decrease in OFT crossing associated with a depressive-like behavior manifested by the increased FST immobility time (Rebai et al. 2021; Bulmus et al. 2022). Indeed, although the HFD group showed a decrease in the number of crossed squares, which represents an index of the vertical locomotion, the same group did not exhibit a significant change in the frequency of rearing, which represents an index of the vertical locomotion, highlighting that the reduced crossing is an emotional phenotype rather than a result of a motor deficit.

The HFD-induced depressive-like behavior was associated with an impairment rats’ performance in the MWM reflecting impairments in the hippocampal-dependent spatial memory (Inostroza et al. 2011). HFD-induced depressive and cognitive deficits have been the focus of a large body of research (Valladolid-Acebes et al. 2013; Boitard et al. 2014; Kesby et al. 2015; Vagena et al. 2019; Yu et al. 2021; Tsai et al. 2022).

The increased serum corticosterone reported in the current study is in line with previous studies revealing HFD-induced activation of the hypothalamo-pituitary-adrenal (HPA) axis, a frequently documented consequence of obesity, which has been correlated to HFD-associated cognitive impairments (Boitard et al. 2014; Janthakhin et al. 2022). The HPA axis represents a neuroendocrine system that regulates the response to stress. While HPA axis activation during acute stress could enhance some aspects of cognitive performance, sustained HPA axis activation could impair neurogenesis and synaptic functions, exhibiting detrimental effects on hippocampal cognitive and affective functions (McEwen 2007; Stephens and Wand 2012).

The current study also reports an HFD-induced increase in hippocampal levels of the pro-inflammatory mediators TNF-α and IL-6. Importantly, adipose tissue-mediated overproduction of TNF-α could drive microglial activation with subsequent production of pro-inflammatory cytokines such as IL-1 and IL-6. This neuroinflammation could elicit the development/progression of neurodegeneration (Erickson et al. 2012; Kang et al. 2016). Moreover, HFD could disrupt the integrity of the blood brain barrier (BBB) with subsequent influx of inflammatory cells into the central nervous system (CNS); obese mice exhibited a 53% increase in infiltration of activated macrophages into the CNS (Drake et al. 2011). Interestingly, research indicates that HFD-induced increases in BBB permeability may be more prominent in the hippocampus compared to other brain areas such as the prefrontal cortex, which may underlie the greater vulnerability of the hippocampus to the detrimental effects of HFD (Kanoski et al. 2010).

BDNF is a neurotrophin that plays critical roles in neuronal survival, growth, and differentiation (Miranda et al. 2019). BDNF exhibits its effects via binding to its high-affinity receptor, TrkB (tropomyosin receptor kinase B), leading to the activation of multiple intracellular signaling pathways, including the PI3K/Akt/mTOR cascade (Li et al. 2022). Upon binding of BDNF to TrkB, PI3K is activated, which catalyzes the conversion of phosphatidylinositol-4,5-bisphosphate (PIP2) to phosphatidylinositol-3,4,5-trisphosphate (PIP3), which acts as a second messenger recruiting AKT to the plasma membrane, where it is activated via phosphorylation at Thr 308 and Ser 473 sites (Tian et al. 2023; Guo et al. 2024). The activated AKT could play multiple vital roles, including amelioration of neuroinflammation by regulating the downstream molecular target nuclear factor-kB (NF-kB) alongside the activation of mTOR via tuberous sclerosis complex (TSC)/Ras homolog enriched in brain (Rheb)-dependent mechanism (Wu et al. 2009; Yan et al. 2019; Zhang et al. 2020; Guo et al. 2024).

The activated mTOR represents a key regulator of cell growth and metabolism; the mTOR complex 1 (mTORC1) is primarily responsible for regulating protein synthesis by phosphorylating p70S6 Kinase 1 (S6K1) and eukaryotic translation initiation factor 4E-binding protein 1 (4EBP1). This regulation is vital for synaptic plasticity, which is essential for learning and memory (Costa-Mattioli et al. 2009; Saxton and Sabatini 2017). Moreover, mTORC1 is a major inhibitory regulator of autophagy (Zhang et al. 2018). Through phosphorylation and inhibition of the autophagy-related protein 13 (ATG13) and the serine/threonine kinase ULK1, mTORC1 could block the early steps of autophagy (Wu et al. 2018; Deleyto-Seldas and Efeyan 2021). Autophagy is a vital cellular process that facilitates the degradation and recycling of damaged organelles and misfolded proteins. This process plays a crucial role in maintaining cellular homeostasis (King 2012; Tang et al. 2021). Nevertheless, excessive uncontrolled autophagic flux can trigger neurotoxicity and neurodegeneration (Dagda et al. 2013).

The current study reports an HFD-induced decrease in hippocampal BDNF, PI3K, p-Akt, and p-mTOR proteins, which was confirmed by the reduction in the gene expression of PI3K, Akt, and mTOR. This was accompanied by an increase in the protein and gene expression of Beclin 1 and LC3, denoting a possible increased autophagic flux. These results come in line with a body of studies. HFD induced a decrease in hippocampal BDNF, which was associated with hippocampal neuroinflammation and insulin resistance (Zhang et al. 2022). Long-term HFD also impaired autophagic flux in mouse hippocampus with increased hippocampal Beclin 1 and LC3B levels (Chen et al. 2022). Notably, decreased PI3K/Akt/mTOR signaling with dysfunctional autophagy has been intensely linked to hippocampal neuronal damage and subsequent depressive-like behaviors and cognitive dysfunction (Shih et al. 2019; Zhang et al. 2021; Xu et al. 2023).

The results of the present study indicate that liraglutide amended the HFD-induced depressive and cognitive deficits. This was accompanied by a decrease in serum corticosterone, denoting a restored normal functioning of the HPA axis with a decrease of hippocampal TNF-α and IL-6. The HFD/Lira group also exhibited an increase in hippocampal BDNF, PI3K, p-Akt, and p-mTOR proteins, with an increase in the gene expression of PI3K, Akt, and mTOR alongside a decrease in the protein and gene expression of Beclin 1 and LC3, inferring a possible inhibition of autophagic flux. This was reflected on the cellular level, where liraglutide ameliorated HFD-induced neurodegenerative changes. Indeed, these favorable effects of liraglutide are consistent with other earlier reports (Wang et al. 2018; Yang et al. 2018; Weina et al. 2018; Kahal et al. 2019; Yao et al. 2021; An et al. 2023; Guan et al. 2023; Seo et al. 2023).

Liraglutide is an acylated GLP-1 agonist indicated for the treatment of diabetes mellitus and as an adjunct to diet regulation and physical exercise for the management of chronic obesity in adults with a BMI ≥30 kg/m^2^ (Cena et al. 2020). Endogenous GLP-1 is secreted within the brain, and its primary source is the preproglucagon neurons in the nucleus tractus solitarius (NTS) (Diz-Chaves et al. 2020). In situ hybridization technique and immunohistochemical studies revealed that GLP-1 receptors (GLP-1R) are widely distributed throughout the brain in the cerebral cortex, hypothalamus, hippocampus, thalamus, caudate nucleus, and medulla oblongata (Farr et al. 2016; Jensen et al. 2018). Notably, fluorescent probe experiments reveal that peripherally administered liraglutide can cross the blood-brain barrier (BBB) and reach the brain either through passive diffusion or via binding to the central GLP-1R (Secher et al. 2014; Dong et al. 2022). Liraglutide’s BBB penetration was associated with stimulation of GLP-1R and induction of neurogenesis in mice brains (Hunter and Hölscher 2012). GLP-1 agonism could mediate a range of physiological processes within the brain, including regulation of the feeding behavior and satiety, the activity of the HPA axis, and the overall physiological response to stress (Diz-Chaves et al. 2020). Interestingly, binding of GLP-1 agonists to the GLP-1 receptor triggers activation of adenosine cyclase with an increase in intracellular cAMP levels with subsequent activation of numerous signaling cascades, including PI3K and its downstream targets (Cheng et al. 2022). Importantly, the liraglutide-mediated activation of the PI3K/Akt/mTOR signaling cascade with subsequent possible inhibition of the exaggerated autophagic flux, impedance of neuroinflammation, and inhibition of neurodegeneration could underlie the favorable antidepressant and pro-cognitive effects exhibited by liraglutide in this work.

## Limitations

One of the major limitations of the study is the absence of experiments using GLP-1 receptor antagonists, such as Exendin (9–39), to confirm the receptor specificity of liraglutide’s effects. Other limitations include the relatively young age of rats at the start of the experiments, the absence of measurements of the p62/SQSTM1 levels and the absence of lysosomal inhibitors (e.g., bafilomycin A1 or chloroquine), which could give a clearer idea about the state of autophagic flux, the limited specificity of some behavioral interpretations, and the incomplete verification of liraglutide’s CNS penetration or target engagement.

## Conclusions

This work indicates the antidepressant and pro-cognitive properties of liraglutide in HFD-exposed rats, which could be mediated through amelioration of the disrupted PI3K/Akt/mTOR signaling activity with a possible impedance of the exaggerated autophagy-mediated neurodegeneration. Indeed, this study highlights that liraglutide is not only effective in the reduction of body weight, but its effects also extend to managing obesity-related psychiatric disorders.

## Data Availability

All data generated or analyzed during this study are available from the corresponding author upon reasonable request.
